# Prediction of beta-turns at over 80% accuracy based on an ensemble of predicted secondary structures and multiple alignments

**DOI:** 10.1186/1471-2105-9-430

**Published:** 2008-10-10

**Authors:** Ce Zheng, Lukasz Kurgan

**Affiliations:** 1 Department of Electrical and Computer Engineering, University of Alberta, Edmonton, AB, Canada

## Abstract

**Background:**

*β*-turn is a secondary protein structure type that plays significant role in protein folding, stability, and molecular recognition. To date, several methods for prediction of *β*-turns from protein sequences were developed, but they are characterized by relatively poor prediction quality. The novelty of the proposed sequence-based *β*-turn predictor stems from the usage of a window based information extracted from four predicted three-state secondary structures, which together with a selected set of position specific scoring matrix (PSSM) values serve as an input to the support vector machine (SVM) predictor.

**Results:**

We show that (1) all four predicted secondary structures are useful; (2) the most useful information extracted from the predicted secondary structure includes the structure of the predicted residue, secondary structure content in a window around the predicted residue, and features that indicate whether the predicted residue is inside a secondary structure segment; (3) the PSSM values of Asn, Asp, Gly, Ile, Leu, Met, Pro, and Val were among the top ranked features, which corroborates with recent studies. The Asn, Asp, Gly, and Pro indicate potential *β*-turns, while the remaining four amino acids are useful to predict non-*β*-turns. Empirical evaluation using three nonredundant datasets shows favorable Q_total_, Q_predicted _and MCC values when compared with over a dozen of modern competing methods. Our method is the first to break the 80% Q_total _barrier and achieves Q_total _= 80.9%, MCC = 0.47, and Q_predicted _higher by over 6% when compared with the second best method. We use feature selection to reduce the dimensionality of the feature vector used as the input for the proposed prediction method. The applied feature set is smaller by 86, 62 and 37% when compared with the second and two third-best (with respect to MCC) competing methods, respectively.

**Conclusion:**

Experiments show that the proposed method constitutes an improvement over the competing prediction methods. The proposed prediction model can better discriminate between *β*-turns and non-*β*-turns due to obtaining lower numbers of false positive predictions. The prediction model and datasets are freely available at .

## Background

Secondary protein structure information provides useful input for a wide range of applications including prediction of solvent accessibility [1], fold type [2], folding rate [3], beta-turns [4], alpha-turns [5], contact order [6], tertiary structure [7], and in fold recognition [8]. The secondary structures include helices, strands, tight turns, bulges, and random coils structures. Helices and strands are repetitive motifs that stabilize the protein structure and which are classified as regular secondary structures. The remaining secondary structures, which are generally designated as random coil regions, are non-repetitive motifs that are regarded as irregular secondary structures [9]. The tight turns can be further divided into *α*-, *β*-, *γ*-, *δ*-, and *π*-turns depending on the number of the constituent residues. The *β*-turns consist of four consecutive residues. The distance between the first, *i*^th^, and the last, *i*^th^+3, residue in a *β*-turn must be less than 7 Å [10,11]. This distance implies a particular geometry of the corresponding backbone, which turns back on itself or, more generally, which corresponds to a change of direction. The *β*-turns are usually described as orienting structure because they orient *α*-helices, *β*-sheets, indirectly defining the topology of proteins. They are one of the most abundant secondary structures, i.e., on average about 25% residues in all protein chains form *β*-turns [12]. The *β*-turns play several significant biological roles in proteins and peptides. They tend to be more solvent exposed than buried and as a result they were found helpful in the context of molecular recognition and in modeling interactions between peptide substrates and receptors [13]. *β*-turns are also involved in the biological activity of peptides as the bioactive structures that interact with other molecules such as receptors, enzymes, or antibodies. Recent years have seen interest in mimicking *β*-turns for the synthesis of medicines [14,15]. Formation of *β*-turns is also a vital stage during the process of protein folding [16]. As a result, development of accurate prediction methods to identify *β*-turns in protein sequences would provide valuable insights and inputs for the fold recognition and drug design.

The *β*-turn prediction methods can be divided into those based on statistics and based on machine learning. Statistical methods utilize probabilities computed using information concerning preferences of individual amino acid types at each position in *β*-turns. They include Chou-Fasman method [10], Thornton's algorithm [17], GORBTURN [18], 1–4 & 2–3 correlation model [19], sequence-coupled model [20], and COUDES method [4]. The first five methods use the sequence as the input, while the COUDES is based on propensities of individual residues augmented with the multiple alignment. The position-specific scoring matrix (PSSM), which is calculated with PSI-BLAST [21], was used to weigh propensities for a given residue, so that all the residues present in the multiple alignment at this position are taken into account. Secondary structure predicted by PSIPRED [22], SSPRO2 [23], and PROF [24] and the flanking residues around the *β*-turn tetrapeptide were also utilized by COUDES to improve the prediction accuracy.

Machine learning based methods include BTPRED [25], BetaTPred2 [26,27], and MOLEBRNN [28] which are based on artificial neural networks (ANN), Kim's method [29] which utilizes *k*-nearest neighbor (kNN), and four methods based on support vector machines (SVMs) [30-33]. BTPRED encodes the sequence using a large window of 11 residues centered over the predicted residue together with secondary structure predicted with PHDsec [34] for the central (with respect to the window) residue to perform predictions. BetaTPred2 is an improved neural network design, in which two networks are used. The MOLEBRNN is based on a bidirectional Elman-type recurrent neural network and uses PSSM profiles as the input. The latter method predicts *β*-turns and their types. In Kim's method, the protein sequence encoded using a window of up to 9 residues is used as the input to kNN, which is combined with a filter that uses secondary structure predicted with PSIPRED for the central residue. The BTSVM method by Pham and colleagues (2003) applies position-specific frequent matrix (PSFM) and PSSM, both computed with PSI-BLAST, to encode input for SVM classifier [31]. The SVM based method proposed by Zhang and colleagues [32] uses PSSM over a 7-residue window and the secondary structure of the central residue predicted by PSIPRED as the input. Both, this method and Kim's method also utilize a filtering stage based on 'state-flipping' rules [25]. The newest SMV-based predictor that was developed by Hu and Li combines the increment of diversity, position conservation scoring function, and secondary structures predicted with PSIPRED to compute the inputs for prediction of *β*-turns and *γ*-turns [33]. Performance of six *β*-turn prediction methods, including Chou-Fasman method, Thornton's method, 1–4 & 2–3 correlation, sequence-coupled method, GORBTURN, and BTPRED was compared using a benchmark database of 426 nonredundant (pairwise-sequence-identity of below 25%) proteins [35]. This dataset was later used to compare and evaluate the subsequently proposed methods and is also adopted in this work.

We observe that although information coming from the sequence (either the sequence itself or the PSSM) was encoded using a window, the existing methods did not use the window when processing information coming from the predicted secondary structure. Moreover, the existing methods apply only one predicted secondary structure at the time, when different predictors are shown to provide complementary predictions [36]. To this end, we combine the PSSM values with information obtained by utilizing an ensemble of four secondary structure prediction methods that was processed using a window to improve the *β*-turn predictions. We also perform feature selection that allows identifying which information (among the predicted secondary structures and PSSM values) is useful for the predictions. Our main goal is to create a prediction model that improves the overall prediction accuracy, Q_total_. The highest reported Q_total _obtained based on the cross-validation on the benchmark dataset of 426 sequences equals 79.8% [33]. Since 25% of residues are *β*-turns, the above result translates in just 4.8/25 = 19% error rate reduction over a naïve method that would classify all residues as not *β*-turns. We note that the percentage of correct predictions among all predicted *β*-turns, Q_predicted_, that is reported for the existing methods ranges between 32.4 and 56%. This means that about half or more of the residues predicted as *β*-turns are in fact not *β*-turns. To this end, our goal is also to provide prediction method characterized by low false positive predictions that would correspond to higher Q_predicted _values, when providing favorable Q_total _values.

## Methods

### Datasets

We apply three different nonredundant protein databases, which were proposed in [4,37], to validate the proposed method. The dataset of 426 protein sequences (denoted by BT426), which was developed by Guruprasad and Rajkumar (2000) [37], is the most widely used benchmark dataset. The two other datasets contain 547 (denoted by BT547) and 823 (denoted by BT823) protein sequences. They were constructed using PDBSELECT list published in June 2000 and October 2003 [38], respectively, by Fuchs and Alix (2005) [4]. The three datasets share several characteristics, such as that the *β*-turns are assigned using PROMOTIF [39], the pairwise sequence identity between any two protein chains is below 25%, the protein structure is determined by X-ray crystallography with at least 2.0 Å resolution, and that each chain contains at least one *β*-turn. The total number of residues in the BT426, BT547, and BT823 datasets equals 96339, 104522, and 150969, respectively. To facilitate computationally expensive parameterization of the classifier and feature selection that were performed in this paper we created another, smaller dataset. We selected three out of the predefined seven folds in the BT426 dataset [35], and in each of these three folds we randomly selected 20% of residues; the corresponding dataset is named BT426-20. We note that the parameterization with the BT426-20 dataset is equivalent to the parameterizations performed by the competing methods, which was based on the original BT426 dataset, i.e., the same division into training and test sets is used.

### Design of the proposed prediction method

**Figure 1:**
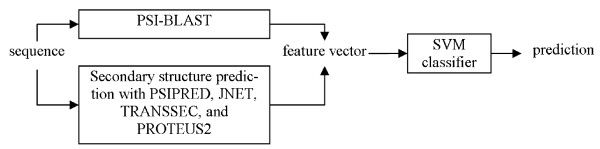
The architecture of the proposed prediction method.

The overall architecture of the proposed system is shown in Figure 1. The protein sequence is converted into a feature vector that incorporates information from the PSSM matrix generated with PSI-BLAST [21] and secondary structure predicted with four prediction methods [36,40,41]. The feature vector, which is computed using a window over the PSSM and the predicted secondary structures that is centered on the predicted residue, is fed into Support Vector Machine classifier to compute the predictions.

### Feature vector

The PSSM indicates whether a given residue in the query sequence is conserved [21]. Since the conservation is usually indicative of the formation of repetitive motifs such as the secondary structures, this information was found useful in prediction of *β*-turns [4,26-28,31-33]. The PSSM is computed using multiple alignment that requires use of a window, and thus the input of the above *β*-turn predictors was also encoded using a window. The PSSM, which is a matrix of 20 × *M *elements, where *M *is the size of the window, was generated with the PSI-BLAST [21] against the NR (nonredundant) NCBI database [42] using default parameters. Similarly as in [32], standard logistic function was used to normalize the PSSM values to <0, 1> interval. A sliding window of seven residues, i.e., *M *= 7, was used to extract features from the PSSM, and the matrix was filled with 0's for positions outside of the sequence, i.e., where the window is located at the sequence termini. The size of 7 is sufficient to account for the coupling effect of residues within *β*-turns since the turns involve 4 residues and thus they should not stretch beyond *i*^th^+3 and *i*^th^-3 positions, where the *i*^th ^position corresponds to the center of the window. The size of 7 or 9 was reported by Shepherd et al. (1999) to achieve the best predictions with BTPRED [25] and the two most recent SVM based methods also apply 7-residue window [32,33].

Four secondary structure prediction methods, PSIPRED v2.5 [8,40], JNET [41], TRANSSEC [36] and PROTEUS2 [36] were employed to produce features. The motivation to use PSIPRED comes from the work of the authors of the COUDES *β*-turn predictor, which concluded that predictions when using PSIPRED were better than when using SSPRO2 and PROF secondary structure prediction methods [4]. This also motivated exclusion of the two latter secondary structure prediction methods. We note that PSIPRED was used to predict *β*-turns in several other methods [4,26,27,29,33]. PROTEUS2 is a recently developed secondary structure prediction method that was selected due to its reported favorable performance when compared with eight competing secondary structure predictors [36]. The main reason to select the other two methods was their unique architectural design that could potentially lead to generation of complementary predictions. TRANSSEC uses two-tier neural network architecture in which the first network is fed with multiple alignment generated with PSI-BLAST and the second network operates on the secondary structure determined via the first network [36]. JNET utilizes a consensus neural network design, in which several different multiple sequence alignment profiles (based on PSI-BLAST and Hidden Markov Models) are combined using a "jury" neural network.

The four secondary structure prediction methods output the 3-state, i.e., helix (H), strand (E), and coil (C), predictions and the confidence score with values between 0 and 9. These values together with the normalized PSSM scores over the 7 residues window were used to compute the following 216 features for each predicted residue:

- *PSSM*_*ij *_is the normalized PSSM value where *i *= 1, 2, ..., 7 denotes the position in the sliding window and *j *= 1, 2, ..., 20 denotes the position in the PSSM (total of 140 features). We assume PSSM values of 0 for the positions in the window that are outside of the sequence.

- *SSP*_*k *_is a binary value denoting the prediction of a given secondary structure method for the central residue, where *SSP *= {PSI (PSIPRED), JNE (JNET), TRA (TRANSSEC), PRO (PROTEUS2)} and *k *= {C, E, H} denotes the predicted secondary structure (total of 4 × 3 = 12 features).

- *SCORE*_*SSP *_= confidence score/10 is the confidence value (division by 10 results in normalization to a unit interval) obtained for the central residue by the *SSP *prediction method (total of 4 features).

- *3PATTERN*_*m*, *k*, *SSP *_is a binary value denoting a specific configuration of the secondary structure predicted with SSP method for the central and the two adjacent residues where *m *= 1, 2, 3, 4 denotes a pattern type. For *m *= 1 and *k *= C the secondary structure prediction would be CCC, and for *m *= 2, 3, and 4 the prediction would be CCx, xCC, and xCx, respectively, where x = {E, H}. We assume coils for the positions outside of the sequence, i.e., when computing at the sequence termini. The total number of these features equals 48 (4 patterns, 3 secondary structures, and 4 *SSP*s). They encode whether the central (predicted) residue is located inside a secondary structure segment or at the interface between two segments.

- *CONTENT*_*k*, *SSP *_is the content of the secondary structure *k *predicted with method *SSP *over a 7 residues window (total of 12 features). The content is defined as the ratio between the number of residues in a given secondary structure and the window size. The window size is shortened when the central residue is at the termini of the sequence.

The first two feature sets are consistent with the inputs used by the competing prediction methods [25-33], except that *SSP*_*k *_is computed for four (instead of one) secondary structure prediction methods. The latter three feature sets are proposed in this paper in an attempt to incorporate window based information concerning the predicted secondary structure and the confidence scores that are provided together with the predictions.

### Prediction method

The support vector machine (SVM) [43] classifier was applied to predict *β*-turns. Given a training set of data point pairs (*x*_*i*_, *c*_*i*_), *i *= 1, 2, ... *n*, where *x*_*i *_denotes the feature vector, *c*_*i *_={-1, 1} denotes binary class label (*β*-turn, non *β*-turn), *n *is the number of training data points, finding the optimal SVM is achieved by solving:

$$\begin{array}{c} \min{\left\|w\right\|}^{2}+C\sum_{i}\xi_{i}\\ \begin{array}{cc} \text{such that} & c_{i}(wz_{i}-b)\geq 1-\xi_{i}\text{ and }1\leq i\leq n \end{array} \end{array}$$

where *w *is a vector perpendicular to *wx *- *b *= 0 hyperplane that separates the two classes, *C *is a user defined complexity constant, *ξ*_*i *_are slack variables that measure the degree of misclassification of *x*_*i *_for a given hyperplane, *b *is an offset that defines the size of a margin that separates the two classes, and *z *= *φ*(*x*) where *k*(*x*, *x*') = *φ*(*x*)·φ(*x*') is a user defined kernel function. The SVM classifier was trained using Platt's sequential minimal optimization algorithm [44], which was optimized by Keerthi et al (2001) [45]. The radial basis functions (RBF) was used as kernel function

k(xi,xi')=e−γ‖x−x'‖2

where *γ *is the user defined constant.

The SVM classification algorithm and feature selection algorithms (see next section) used to build and test our prediction method were implemented in Weka [46]. The selection of the classifier and the kernel type was motivated by the best *β*-turn predictions reported to date on the BT426 dataset that were obtained using RBF-kernel based SVM classifier [32,33]. The proposed classifier is parameterized, i.e., the values of *C *and *γ *are selected, based on the procedure described in the next section.

### Features selection and parameterization of SVM

Since total of 216 features were generated for each predicted residue and we expect that some of them would not contribute to the prediction, a feature selection was performed to reduce the dimensionality. This allows for reduction of the time necessary to compute the prediction model and for finding and discussing which of the proposed features are related to the prediction of *β*-turns.

Three feature selection approaches were employed. The first, hybrid approach combines the Information-Gain (IG) [47] and the Chi-Squared (CHI) [48] feature selection methods. The two methods were selected based on their successful application in [49] and [50], respectively, where they were used to rank protein sequence based features. We also applied a filter-based feature selection method which removes features based on the inconsistency criterion [51] and a wrapper-based algorithm [52] that applies flexible Naïve Bayes classifier [53]. In the case of the filter-based and the wrapper-based methods, we used best first search that starts with empty set of features to search through possible subsets of features. The selection of this search method and the selection of the flexible Naïve Bayes to evaluate feature sets in the wrapper-based method were motivated by their good scalability, which was necessary due to the large size of the datasets, i.e., the search method is linear with respect to the number of features and the Naïve Bayes is linear with respect to the number of data points.

In the hybrid feature selection (which leads to favorable quality of predictions, see "Comparison of different feature selection strategies" Section), we used two different selection methods to reduce bias introduced by each of the methods. In both algorithms, each feature is ranked based on its merit (measured with information gain in IG and the value of the chi-squared statistic in CHI), and next they were sorted by their average rank across the two algorithms. The measurement of the merit for the two selection methods is defined below.

IG measures the decrease in entropy when a given feature is used to group values of another feature. The entropy of a feature *X *is defined as:

$$H(X)=-\sum_{i}P(x_{i})\log_{2}(P(x_{i}))$$

where {*x*_*i*_} is a set of values of *X *and *P*(*x*_*i*_) is the prior probability of *x*_*i*_. The conditional entropy of *X*, given another feature *Y *(in our case the *β*-turn/non *β*-turn labels) is defined as:

$$H(X|Y)=-\sum_{j}P(y_{j})\sum_{i}P(x_{i}|y_{j})\log_{2}(P(x_{i}|y_{j}))$$

where *P*(*x*_*i*_|*y*_*j*_) is the posterior probability of *X *given the value *y*_*i *_of *Y*. The amount by which the entropy of *X *decreases reflects additional information about *X *provided by *Y *and is called information gain

*IG*(*X*|*Y*) = *H*(*X*) - *H*(*Y*|*Y*)

According to this measure, *Y *has stronger correlation with *X *than with *Z *if *IG*(*X*|*Y*) > *IG*(*Z*|*Y*).

CHI method is based on a common statistical test that measures divergence from the expected distribution assuming that the occurrence of a given feature is independent of the class value. Let *X *be a discrete random variable (which corresponds to a feature in this paper) with *m *= 2 possible outcomes *x*_1 _= *β*-turn, *x*_2 _= non *β*-turn, and with probability of each outcome *P*(*X *= *x*_*i*_) = *p*_*i*_. Pearson-chi-squared statistic is defined as:

$$\chi^{2}=\sum_{i=1}^{m}\frac{{\left(n_{i}-np_{i}\right)}^{2}}{np_{i}}$$

where *n*_*i *_is the number of instances which will result the outcome *x*_*i*_. A feature that obtains higher *χ *value receives lower rank.

To avoid overfitting, the 216 features were ranked on the BT426 dataset using 7-fold cross validation. In the case of the hybrid method, the average ranks (average over the ranks produced by IG and CHI methods) were computed for each of the 7 training datasets separately, and next they were averaged over the 7 datasets. After the features are ranked, we performed *β*-turn prediction in order to decide how many of the features should be kept. This was performed in two steps. First, we selected the top 50 features to perform parameterization of the SVM classifier. Next, we used the parameterized SVM to select the desired number of features.

**Figure 2:**
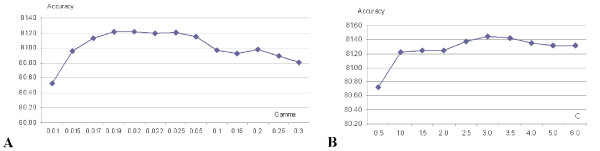
Parameterization of SVM based on 3 fold cross-validation test with top 50 features on the BT426-20 dataset; panel A shows search for optimal *γ *when *C *= 1; panel B shows search for optimal C using optimal values of *γ *= 0.0186; the *x*-axis shows values of parameters, while *y*-axis shows the corresponding accuracy (Q_total_) of *β*-turn prediction.

In step 1 we perform greedy search for optimal *C *and *γ *parameters:

- We freeze *C *= 1 and find the optimal *γ *based on 3-fold cross validation on the BT426-20 dataset using the top 50 features, see Figure 2A. We observe that highest accuracy (Q_total_) is achieved for *γ *equal 0.0186 or 0.02. The optimal *γ *= 0.0186 is the same as the value used in [32].

- Next, we use the *γ *= 0.0186 and we optimize *C*, see Figure 2B. The best accuracy is achieved for *C *= 3.

We did not apply the grid-based parameterization, which could potentially lead to better parameters, since this would be computationally expensive and since Figure 2 shows that the SVM is not sensitive to the parameters, i.e., the Q_total _values change by only up to 0.7% by varying *γ *and *C*.

**Figure 3:**
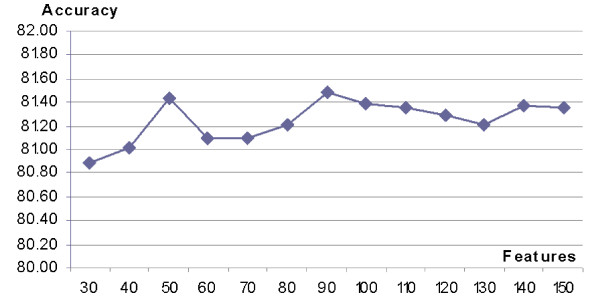
Selection of the desired number of features based on 3 fold cross-validation test on the BT426-20 dataset using SVM with *C *= 3 and *γ *= 0.0186; the *x*-axis shows the number of selected top features, while *y*-axis shows the corresponding accuracy (Q_total_) of *β*-turn prediction.

In the second step, the SVM classifier with the optimized parameters (*C *= 3 and *γ *= 0.0186) was used to select the desired number of the top ranked features. The selection was again based on 3-fold cross validation on the BT426-20 dataset, see Figure 3. The results show that selection of the top 90 features provides the best accuracy, although we note that the differences are relatively small. This indicates that only a small fraction of the original features is necessary to provide accurate *β*-turn predictions.

Since the wrapper- and filter-based feature selections directly select a set of features, rather than ranking the features as it is done by the hybrid method, there was no need to decide how many features should be kept. Instead, each method was used to select a set of features for each of the 7 training folds from the BT426 dataset, and next these features were combined together. This resulted in selection of 54 and 39 features in the case of the filter-based and wrapper-based algorithms, respectively.

These two feature sets were used with SVM parameterized using the above procedure where the top 50 ranked features were applied. We did not re-parameterize the SVM for the other two sets of features because the above parameterization shows that the SVM is not sensitive to the parameters, the 50 features used in the parameterization overlap with features used in the other two sets, and since such parameterization is computationally expensive.

### Evaluation procedure and measures

7-fold cross validation was used to perform tests on the three datasets (BT426, BT547 and BT823). The folds were created by randomly selecting equal number of sequences. The quality of prediction is evaluated using four measures, Q_total_, Q_predicted_, Q_observed_, and MCC. This is consistent with the test procedures and measures applied to evaluate modern competing methods [4,25-33,35].

Given that TP (true positives) is the number of correctly classified *β*-turn residues, TN (true negatives) is the number of correctly classified non-*β*-turn residues, FP (false positives) if the number of non-*β*-turn incorrectly classified as *β*-turn residues, and FN (false negatives) is the number of *β*-turn incorrectly classified as non-*β*-turn residues, Q_total _(prediction accuracy) is defined as the percentage of correctly classified residues.

$$Q_{total}=\frac{TP+TN}{TP+TN+FP+FN}\times 100$$

Probability of correct prediction, Q_predicted_, is the percentage of correctly predicted *β*-turns among the predicted *β*-turns

$$Q_{predicted}=\frac{TP}{TP+FP}\times 100$$

Sensitivity or coverage, Q_observed_, is the percentage of correctly predicted *β*-turns among the observed (true) *β*-turns

$$Q_{observed}=\frac{TP}{TP+FN}\times 100$$

We observe that approximately 24% to 24.6% *β*-turn residues are observed in the three datasets, and therefore Q_total _= 75% (baseline prediction accuracy) could be obtained by merely regarding all residues as non *β*-turns. Therefore, Q_total _values can result in misleading information (overestimation of predictive performances), which was pointed out in [4,25,54]. This is especially evident when TN values are large, compared to TP, FN, and FP values. As a result Matthews Correlation Coefficient (MCC) is computed

$$MCC=\frac{TP\times TN-FP\times FN}{\sqrt{\left(TP+FP)\times (TP+FN)\times (TN+FP)\times (TN+FN\right)}}\times 100$$

The value of MCC is confined to <-1,1> interval. If the MCC value is close to 0 then the prediction method is not better than a random classification. Higher MCC value corresponds to better performance of the prediction method.

We also report receiver-operator characteristics (ROC) curves that present a graphical plot of the TP rate = Q_*observed *_against FP rate = FP/(FP + TN).

## Results and discussion

### Comparison of different feature selection strategies

**Figure 4:**
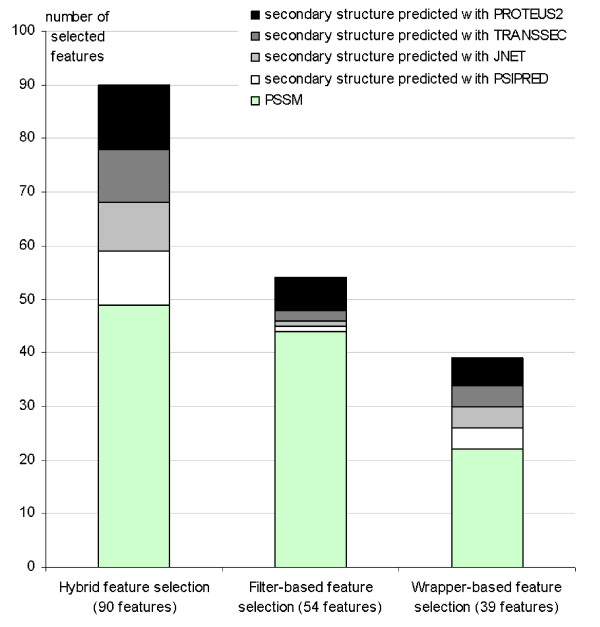
Comparison of three feature sets generated by using the hybrid, the filter-based, and the wrapper-based feature selection methods.

The three employed feature selection methods produce different feature sets which are summarized in Figure 4. In all three cases majority of the features are based on PSSM. Among the features that are computed from the predicted secondary structures, the largest fraction is derived from the structures predicted by PROTEUS2. We also observe that all four predicted secondary structures are used to derive features in each of the feature sets. This consistency supports the need for multiple predicted secondary structures.

**Figure 5:**
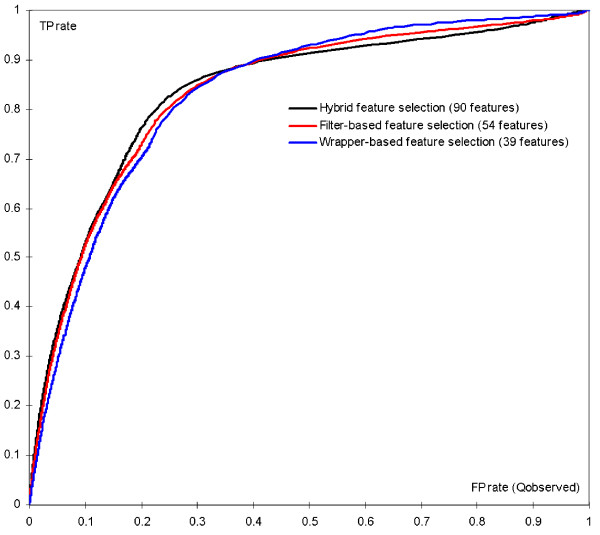
T**he ROC curve (TP rate vs. FP rate) for the prediction of *β*-turns based on the 7-fold cross validation on the BT426 datasets when using three sets of features, which were generated using the hybrid, the filter-based, and the wrapper-based feature selection methods.**

Each of the three set of features was used as the input to the optimized SVM classifier with *C *= 3 and *γ *= 0.0186 to perform prediction of *β*-turns. The evaluation of the quality of these predictions was performed based on 7-fold cross validation on the BT426 datasets, see Table 1. The Table shows that usage of the 90 features selected using the hybrid methods results in the highest MCC value. These features also lead to the highest Q_observed _and Q_total_. At the same time, we note that the features selected using the filter-based method correspond to the highest Q_predicted _value. We observe that the differences are relatively small as all three designs provide the accuracy which is close to 80%. The ROC curves shown in Figure 5 allow for a more detailed analysis of the differences. The 90 features generated by the hybrid method are shown to provide favorable prediction quality for low values of FT rate (up to 0.35), while the 39 features based sequence representation developed with wrapper-based method provides higher TP rate for the higher values of the FP rate. The most interesting part of the curves corresponds to relative high TP rates that are coupled with low values of FP rate. Since the ratio of positive to negative samples is 1:4, the ratio between the true positives and false positives would be even at FP rate of approximately 0.25. For higher FP rate, the number of false positives would be higher than the number of true positives. As a result, we conclude that the 90 features provide better predictions than the other two feature sets and this feature set is used to build the proposed prediction method.

**Table 1:** Performance comparison between the predictions performed using the three sets of features that were generated using the hybrid, the filter-based, and the wrapper-based feature selection methods

**Feature selection method**	**Q**_total_	**Q**_predicted_	**Q**_observed_	**MCC**
Hybrid method (90 features)	80.9	62.7	55.6	0.47
Filter-based (54 features)	80.7	64.1	49.5	0.44
Wrapper-based (39 features)	79.6	60.2	51.0	0.42

The tests are based on 7 fold cross validation on the BT426 dataset

### Comparison with competing prediction methods

The 90 selected features and SVM with *C *= 3 and *γ *= 0.0186 were applied in the proposed prediction model. The 7-fold cross validation test results on the BT426 dataset are summarized and compared with over a dozen of competing methods in Table 2; the results are organized in the descending order by the values of MCC.

**Table 2:** Performance comparison between the proposed and the competing methods based on the 7 fold cross validation test on the BT426 dataset

**Prediction method [reference]**	**Q**_total_	**Q**_predicted_	**Q**_observed_	**MCC**
This paper	80.9	62.7	55.6	0.47
SVM [33]	79.8	55.6	68.9	0.47
MOLEBRNN [28]	77.9	53.9	66.0	0.45
SVM (multiple alignment) [32]	77.3	53.1	67.0	0.45
BTSVM [31]	78.7	56.0	62.0	0.45
BETATPRED2 (multiple alignment) [26,27]	75.5	49.8	72.3	0.43
COUDES (*ψ*_threshold _= 0 for PSSM) [4]	74.8	48.8	69.9	0.42
COUDES (*ψ*_threshold _= -100 for PSSM) [4]	75.5	49.8	66.6	0.41
SVM (single sequence) [32]	74.8	49.1	67.9	0.41
BETATPRED2 (single sequence) [26,27]	74.3	48.4	71.2	0.41
KNN [29]	75.0	46.5	66.7	0.40
BTPRED^1 ^[25]	74.9	55.3	48.0	0.35
BTPRED [25,35]	74.4	48.3	57.3	0.35
Chou-Fasman [10,35]	65.2	37.6	63.5	0.26
Thornton [17,35]	68.0	38.6	52.4	0.23
GORBTURN [18,35]	70.5	39.3	37.3	0.19
1–4 & 2–3 correlation [19,35]	59.1	32.4	61.9	0.17
Sequence coupled [20,35]	53.3	32.4	72.8	0.17

^1 ^Results reported on a different dataset with 300 chains.

Table 2 shows that the proposed method achieves the highest prediction accuracy Q_total _= 80.9%, Q_predicted _= 62.7%, and MCC = 0.47 on the BT426 dataset. Our prediction results in 1.1% higher total accuracy than the Q_total _of the best existing prediction method [33]. We emphasize that this difference is relatively large when considering that the baseline accuracy equals 75%, i.e., our method provides 5.9/25 = 24% error rate reduction while the second best method provides 19% error rate reduction. The Q_predicted _of the proposed method is higher by 6.7% than the Q_predicted _of the BTSVM [31] and by 7.1% when compared with the best existing method by Hu and Li [33]. Higher Q_predicted _values mean that a larger fraction of the predicted *β*-turns are in fact *β*-turns (false positive numbers are lower). This indicates that the proposed prediction model can better discriminate between *β*-turns and non *β*-turns when compared with the competing methods. At the same time, the Q_observed _value obtained by the proposed method shows that over 55% of actual *β*-turns were correctly predicted. We note that our Q_observed _value is 11.4% lower than the Q_observed _reported in [32] and 13.3% lower than the Q_observed _reported in [33]. The increase of Q_predicted _values as a trade-off for decreased Q_observed _values is due to the usage of the predicted secondary structure. This is since the proposed method would not predict *β*-turns inside the predicted helices and strands, which reduces the number of false positives; this observation is consistent with the conclusions in [4]. At the same time, if the helix/strand prediction is incorrect, i.e., the actual structure is a *β*-turn, than our prediction method would most likely produce a false negative prediction. We show that in spite of this trade-off, the overall accuracy is improved, i.e., both Q_total _and MCC values are the highest in the case of the proposed method.

We investigate the impact of the proposed features extracted from the predicted secondary structure on the prediction quality. When excluding the 49 features that are based on PSSM (out of 90 features used in the proposed method), the predictions performed with the remaining 41 secondary-structure features are characterized by Q_total _= 80.3%, Q_predicted _= 60.9%, Q_observed _= 56.3, and MCC = 0.46. Although removal of the PSSM features results in slightly worse predictions, we observe that the features computed from the predicted secondary structures provide valuable input. We also experimented with the design in which only one of the four secondary structure predictions is used. We used output of the PROTEUS2 that is characterized by a favorable performance when compared with the other three secondary structure prediction methods [36]. The predictions that use only the 49 PSSM based features and 12 features generated from secondary structure predicted by PROTEUS result in Q_total _= 80.5%, Q_predicted _= 61.3%, Q_observed _= 56.8, and MCC = 0.46. We observe that these results are again slightly worse than the results obtained with the proposed method; the usage of all 90 features improves Q_total_, Q_predicted_, and MCC. The simplified architecture that uses only one predicted secondary structure is equivalent to the design of the recent method by Zhang and colleagues [32]. We note that our results are better than the results of this method, i.e., Q_total _= 80.5% vs. Q_total _= 77.3%, which stems from using different secondary structure prediction method and the novel features. Our method uses features computed from a window over the predicted secondary structure, while the Zhang's method uses only the secondary structure of the predicted residue.

Results obtained based on the 7-fold cross validation with the BT547 and BT823 datasets are given in Table 3. They show that for the BT547 dataset the proposed method obtains 3.9% higher Q_total_, 2% higher MCC, 14% better Q_predicted_, and 16% lower Q_observed _when compared with the second-best SVM based method [33]. Similarly, for the BT823 dataset, our method obtains 3.8% and 7.8% increase in Q_total _and Q_predicted_, respectively, and 17.7% decrease in Q_observed_. We observe that the proposed method obtains consistent (similar) quality of predictions for all three datasets. The Q_total _values range between 80.5% and 80.9%, Q_predicted _values range between 60.8% and 62.7%, Q_observed _between 54.2% and 55.6%, and MCC between 0.45 and 0.47, see Tables 2 and 3. This rules out possibility of overfitting the BT426 dataset due to the performed design.

**Table 3:** Performance comparison between the proposed method, the COUDES method, and the most recent SVM-based method by Hu and Li

**Prediction method [reference]**	**Dataset**	**Q**_total_	**Q**_predicted_	**Q**_observed_	**MCC**
This paper	BT547	80.5	61.6	54.2	0.45
COUDES [4]		74.6	48.7	70.4	0.42
SVM [33]		76.6	47.6	70.2	0.43

This paper	BT823	80.6	60.8	54.6	0.45
COUDES [4]		74.2	47.5	69.6	0.41
SVM [33]		76.8	53.0	72.3	0.45

The tests are based on the 7 fold cross validation test on the BT547 and BT823 datasets

### Analysis of the proposed feature based sequence representation

The proposed method applies 90 features, which constitutes 86% reduction of the dimensionality of the input vector when compared with the method by Hu and Li (2008) that applies 628 features (140 values from PSSM using 7-residue window, 7 conservation scores, 3 features to encode the PSIPRED [8,40] predicted secondary structure, and 478 features that are processed by the increment of diversity algorithm). The dimensionality reduction of 62% and 37% is achieved when compared with the two third-best performing, i.e., with the third best MCC value, SVM-based predictors by Pham and colleagues (2003), which uses 240 features (PSSM values using 12 residues window) [31], and the method by Zhang and coworkers (2005) that applies 143 features (140 PSSM values using 7 residues window and 3 features to encode the PSIPRED predicted secondary structure for the predicted residue), respectively.

The top 10 ranked features that were used to implement the proposed prediction method are shown in Table 4. We observe that 6 out of 10 features are the newly proposed features, which are based on the sliding window over the secondary structure predicted by three prediction methods, PROTEUS2 [36], PSIPRED [8,40], and JNET [41]. The remaining 4 features concern the predicted secondary structure for the predicted residue. 7 out of 10 features are computed using the predicted coils, while the remaining 3 concern predicted helices. The former is likely due to *β*-turns being a coil subtype and due to the favorable accuracy that the secondary structure prediction methods obtain for coils and helices when compared with the accuracy for strands [55,56]. We note that 6 out of 10 features are extracted from the predictions performed with PROTEUS2, which confirms high quality of this method. Finally, Table 4 reveals the top 10 features were based on the predicted secondary structure, i.e., none of them was based on PSSM [21], which signifies the novelty of the proposed approach.

**Table 4:** The top 10 features used in the proposed prediction method

**Rank**	**Abbreviation**	**Details**
1	*CONTENT*_*C*, *PRO*_	Content of coils in window predicted with PROTEUS2
2	*PRO*_*C*_	Coil predicted for the central residue with PROTEUS2
3	*CONTENT*_*C*, *PSI*_	Content of coils in window predicted with PSIPRED
4	*CONTENT*_*C*, *JNE*_	Content of coils in window predicted with JNET
5	*PSI*_*C*_	Coil predicted for the central residue with PSIPRED
6	*CONTENT*_*H*, *PRO*_	Content of helices in window predicted with PROTEUS2
7	*JNE*_*C*_	Coil predicted for the central residue with JNET
8	*3PATTERN*_1, *C*, *PRO*_	Pattern CCC for structure predicted with PROTEUS2
9	*3PATTERN*_1, *H*, *PRO*_	Pattern HHH for structure predicted with PROTEUS2
10	*PRO*_*H*_	Helix predicted for the central residue with PROTEUS2

Furthermore, we analyzed the composition of the top 90 selected features. The highest scoring *PSSM*_*ij *_feature was ranked 32 and total of 49 out of 90 features were derived from the PSSM. Among the remaining 41 features, 12 concern structure predicted for the central residue (*SSP*_*k*_), 3 concern the prediction confidence score for the central residue (*SCORE*_*SSP*_), 14 are based on the 3 residue window pattern over the predicted secondary structure (*3PATTERN*_*m*, *k*, *SSP*_), and 12 concern secondary structure content over the 7 residue window (*CONTENT*_*k*, *SSP*_). This shows that all proposed feature sets were utilized to perform the prediction.

Table 5 summarizes the features computed from the predicted secondary structure. It shows that each of the four secondary structure predictions was used to compute similar number of features. Two sets of features, the secondary structure of the central residue (*SSP*_*k*_) and the secondary structure content (*CONTENT*_*k*, *SS*_), were fully included in the selected 90 features, i.e., features computed with all four prediction methods were selected. We note that only 5 out of 12 patterns for the 3 residue window over the predicted secondary structure were utilized, namely CCC, EEE, HHH, CCx, and xCC. The former three patterns concern residues that are inside of a predicted secondary structure segment and they were computed from all four predicted secondary structures. The CCC pattern is likely positively correlated with the prediction of *β*-turns, while the remaining two patterns are likely negatively correlated with the *β*-turns (or positively correlated with non *β*-turns). The latter two patterns (CCx and xCC) were computed from the structure predicted with PROTEUS2, and they concern residues located on the interface between a coil and another secondary structure.

**Table 5:** Summary of features computed from the predicted secondary structure for the four employed prediction methods;√ denotes that a given feature was used, while – denotes a feature that was not selected

**Feature Set**		**secondary structure prediction methods**			
		
		PSIPRED	JNET	TRANSSEC	PROTEUS2
*SSP*_*k*_	*C*	√	√	√	√
	E	√	√	√	√
	H	√	√	√	√

*SCORE*_*SSP*_		√	*-*-	√	√

*3PATTERN*_*m*, *k*, *SSP*_	CCC	√	√	√	√
	CCx	*-*-	*-*-	*-*-	√
	xCC	*-*-	*-*-	*-*-	√
	EEE	√	√	√	√
	HHH	√	√	√	√

*CONTENT*_*k*, *SSP*_	C	√	√	√	√
	E	√	√	√	√
	H	√	√	√	√

Total		10	9	10	12

**Figure 6:**
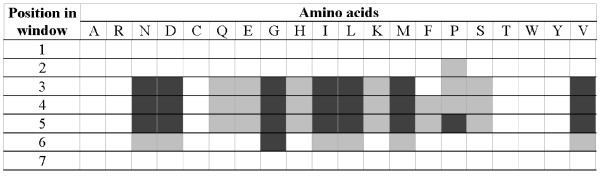
**PSSM features selected to perform *β*-turn prediction.** The rows correspond to positions in the sliding window and columns correspond to the 20 amino acids (depth of the matrix); dark grey denotes selected *PSSM*_*ij *_features that were ranked 31–60, light grey denotes *PSSM*_*ij *_features ranked 61–90, and while cells denote features that were not selected.

Figure 6 summarizes the PSSM derived features that were used to perform *β*-turn predictions. The white cells denote features that were not selected while the shaded cells denote the selected features (darker shading corresponds to higher ranked features). 23 PSSM features were ranked 31 to 60, and the remaining 26 PSSM features were ranked in the bottom 30 positions. We observe that none of the features at positions 1 and 7 (that correspond to the edges of the sliding window) were used, which suggests that window of size 5 over the PSSM matrix would be sufficient for the prediction. We hypothesize that these two positions are no longer necessary, when compared with the results in [32,33], due to the additional information that is encoded from the predicted secondary structures. Figure 6 shows that PSSM values corresponding to Ala (A), Arg (R), Cys (C), Thr (T), Trp (W), and Tyr (Y) were not found useful for the prediction. At the same time, Asn (N), Asp (D), Gly (G), Ile (I), Leu (L), Met (M), Pro (P), and Val (V), were among the top ranked PSSM features. These findings are supported by existing research. The overall potential of amino acids to form *β*-turns was initially evaluated in [57] and than it was recomputed (based on a larger dataset) in [37]. The former study shows that Asn, Asp, Gly, and Pro are characterized by high potential to form *β*-turns, while Ile, Leu, Met, Val, and Trp are characterized by the lowest potential [57]. Similar conclusions, i.e., high potential for Asn, Asp, Gly, and Pro, and low potential for Ile, Leu, Met, and Val, were presented in [37]. We observe that the eight of the above amino acids were found as the most useful to implement the proposed prediction method. Some of them (Asn, Asp, Gly, and Pro) would be useful to indicate potential *β*-turns, while the remaining four (Ile, Val, Leu, and Met) would be useful to predict non *β*-turns. As shown by Pham and colleagues (2003), the Ala, Ile, and Leu tend to prevent formation of *β*-turns, while Asp supports the formation of *β*-turns [31]. This also agrees with our results that show that PSSM values of Ile, Leu, and Asp are among the top ranked PSSM features.

## Conclusion

We propose a novel method for the prediction of *β*-turns that uses features extracted from a window over the three-state secondary structure predicted by an ensemble of four methods. We use feature selection to reduce the dimensionality of the proposed feature vector. The proposed feature set is smaller by 37 to 86% when compared with the best performing competing methods. The analysis of the selected features reveals that:

- information from all four predicted secondary structures is useful for the prediction of *β*-turns,

- the most useful information extracted from the predicted three-state secondary structure are the structure of the predicted residue, content of the secondary structures over 7 residues window centered on the predicted residue, and features that indicate whether the predicted residue is inside a secondary structure segment,

- PSSM values of Asn (N), Asp (D), Gly (G), Ile (I), Leu (L), Met (M), Pro (P), and Val (V), were among the top ranked features; similar conclusions, i.e., high potential of Asn, Asp, Gly, and Pro, and low potential of Ile, Leu, Met, and Val, to form *β*-turns were shown in [37,57]. In our case, Asn, Asp, Gly, and Pro are likely associated with potential *β*-turns, while the remaining four amino acids predict non *β*-turns.

Empirical evaluation using three nonredundant datasets shows that our predictions provide favorable Q_total_, Q_predicted_, and MCC values when compared with over a dozen of modern competing methods. Our method is the first to break the 80% Q_total _barrier and is characterized by 80.9% Q_total_, 0.47 MCC, and Q_predicted _higher by over 6% when compared with the second best method using a benchmark dataset of 426 nonredundant sequences. This shows that our method not only constitutes an improvement over the competition, but also that the proposed prediction model can better discriminate between *β*-turns and non *β*-turns since it generates lower numbers of false positive predictions.

## Abbreviations

ANN: artificial neural networks; CHI: Chi-Squared; FN: false negatives; FP: false positives; IG: Information-Gain; kNN: k-nearest neighbor; MCC: Matthews Correlation Coefficient; PDB: Protein Data Bank; PSFM: position specific frequent matrix; PSSM: position specific scoring matrix; ROC: receiver-operator characteristics; SVM: Support Vector Machine; TN: true negatives; TP: true positives.

## Authors' contributions

CZ helped with the preparation of the datasets, computed the features, performed feature selection, generated the prediction model, performed experimental comparison, and helped with evaluation of the results. LK contributed to the conception and design of the prediction method, helped with the preparation of the datasets, designed the experimental study, helped with the evaluation of the results, prepared the revision, drafted the manuscript, and coordinated the project. Both authors have corrected and approved the manuscript.

## References

[B1] Garg A, Kaur H, Raghava GP (2005). Real value prediction of solvent accessibility in proteins using multiple sequence alignment and secondary structure. Proteins.

[B2] Chen K, Kurgan L (2007). PFRES: Protein Fold Classification by Using Evolutionary Information and Predicted Secondary Structure. Bioinformatics.

[B3] Ivankov DN, Finkelstein AV (2004). Prediction of protein folding rates from the amino acid sequence-predicted secondary structure. Proc Nat Acad Sci USA.

[B4] Fuchs PF, Alix AJ (2005). High accuracy prediction of *β*-turns and their types using propensities and multiple alignments. Proteins.

[B5] Wang Y, Xue Z, Xu J (2006). Better prediction of the location of alpha-turns in proteins with support vector machine. Proteins.

[B6] Song J, Burrage K (2006). Predicting residue-wise contact orders in proteins by support vector regression. BMC Bioinformatics.

[B7] Kim DE, Chivian D, Baker D (2004). Protein structure prediction and analysis using the Robetta server. Nucleic Acids Res.

[B8] McGuffin LJ, Bryson K, Jones DT (2000). The PSIPRED protein structure prediction server. Bioinformatics.

[B9] Richardson JS (1981). The anatomy and taxonomy of protein structure. Adv Protein Chem.

[B10] Chou PY, Fasman G (1974). Conformational parameters for amino acids in helical, *β*-sheet and random coil regions calculated from proteins. Biochemistry.

[B11] Chou KC (2000). Prediction of tight turns and their types in proteins. Anal Biochem.

[B12] Kabsch W, Sander C (1983). Dictionary of protein secondary structure: Pattern recognition of hydrogen-bonded and geometrical features. Biopolymers.

[B13] Rose GD, Gierasch LM, Smith JA (1985). Turns in peptides and proteins. Adv Protein Chem.

[B14] Müller G, Hessler G, Decornez HY (2000). Are beta-turn mimetics mimics of beta-turns?. Angew Chem Int Ed Engl.

[B15] Kee KS, Jois SD (2003). Design of beta-turn based therapeutic agents. Curr Pharm Des.

[B16] Takano K, Yamagata Y, Yutani K (2000). Role of amino acid residues at turns in the conformational stability and folding of human lysozyme. Biochemistry.

[B17] Wilmot CM, Thornton JM (1988). Analysis and prediction of the different types of *β*-turns in proteins. J Mol Biol.

[B18] Wilmot CM, Thornton JM (1990). *β*-Turns and their distortions: a proposed new nomenclature. Protein Eng.

[B19] Zhang CT, Chou KC (1997). Prediction of beta-turns in proteins by 1–4 & 2–3 correlation model. Biopolymers.

[B20] Chou KC (1997). Prediction of beta-turns. J Peptide Res.

[B21] Altschul SF, Madden TL, Schaffer AA, Zhang J, Zhang Z, Miller W, Lipman DJ (1997). Gapped BLAST and PSI-BLAST: a new generation of protein database search programs. Nucleic Acids Res.

[B22] Jones DT (1999). Protein secondary structure prediction based on position-specific scoring matrices. J Mol Biol.

[B23] Pollastri G, Przybylski D, Rost B, Baldi P (2002). Improving the prediction of protein secondary structure in three and eight classes using recurrent neural networks and profiles. Proteins.

[B24] Ouali M, King RD (2000). Cascaded multiple classifiers for secondary structure prediction. Protein Sci.

[B25] Shepherd AJ, Gorse D, Thornton JM (1999). Prediction of the location and type of *β*-turns in proteins using neural networks. Protein Sci.

[B26] Kaur H, Raghava GPS (2003). Prediction of *β*-turns in proteins from multiple alignment using neural network. Protein Sci.

[B27] Kaur H, Raghava GPS (2004). A neural network method for prediction of *β*-turn types in proteins using evolutionary information. Bioinformatics.

[B28] Kirschner A, Frishman D (2008). Prediction of beta-turns and beta-turn types by a novel bidirectional Elman-type recurrent neural network with multiple output layers (MOLEBRNN). Gene.

[B29] Kim S (2004). Protein *β*-turn prediction using nearest-neighbor method. Bioinformatics.

[B30] Cai YD, Liu XJ, Xu XB, Chou KC (2002). Support vector machines for the classification and prediction of beta-turn types. J Peptide Sci.

[B31] Pham TH, Satou K, Ho TB (2003). Prediction and analysis of beta-turns in proteins by support vector machine. Genome Inform.

[B32] Zhang Q, Yoon S, Welsh WJ (2005). Improved method for predicting *β*-turn using support vector machine. Bioinformatics.

[B33] Hu X, Li Q (2008). Using support vector machine to predict beta- and gamma-turns in proteins. J Comput Chem.

[B34] Rost B, Sander C (1994). Combining evolutionary information and neural networks to predict protein secondary structure. Proteins.

[B35] Kaur H, Raghava GPS (2002). An evaluation of *β*-turn prediction methods. Bioinformatics.

[B36] Montgomerie S, Sundararaj S, Gallin WJ, Wishart DS (2006). Improving the accuracy of protein secondary structure prediction using structural alignment. BMC Bioinformatics.

[B37] Guruprasad K, Rajkumar S (2000). *β*-and *γ*-turns in proteins revisited: a new set of amino acid dependent positional preferences and potential. J Biosci.

[B38] Hobohm U, Sander C (1994). Enlarged representative set of protein structures. Protein Sci.

[B39] Hutchinson EG, Thornton JM (1996). PROMOTIF-a program to identify and analyze structural motifs in proteins. Protein Sci.

[B40] Bryson K, McGuffin LJ, Marsden RL, Ward JJ, Sodhi JS, Jones DT (2005). Protein structure prediction servers at University College London. Nucl Acids Res.

[B41] Cuff JA, Barton GJ (2000). Application of multiple sequence alignment profiles to improve protein secondary structure prediction. Proteins.

[B42] Wheeler DL, Church DM, Edgar R, Federhen S, Helmberg W, Madden TL, Pontius JU, Schuler GD, Schriml LM, Sequeira E, Suzek TO, Tatusova TA, Wagner L (2004). Database resources of the National Center for Biotechnology Information: update. Nucl Acids Res.

[B43] Vapnik V (1999). The Nature of Statistical Learning Theory.

[B44] Platt J, Schoelkopf B, Burges C, Smola A (1998). Fast training of support vector machines using sequential minimal optimization. Advances in kernel methods – support vector learning.

[B45] Keerthi SS, Shevade SK, Bhattacharyya C, Murphy KRK (2001). Improvements to Platt SMO Algorithm for SVM Classifier Design. Neural Computation.

[B46] Witten I, Frank E (2005). Data Mining: Practical machine learning tools and techniques.

[B47] Yu L, Liu H (2003). Feature selection for high-dimensional data: a fast correlation-based filter solution. Proceedings of the 10th International Conference on Machine Learning.

[B48] Forman G (2003). An Extensive Empirical Study of Feature Selection Metrics for Text Classification. J Machine Learning Research.

[B49] Chen K, Kurgan L, Ruan J (2007). Prediction of flexible/rigid regions from protein sequences using k-spaced amino acid pairs. BMC Struct Biol.

[B50] Chen K, Jiang Y, Du L, Kurgan L (2008). Prediction of integral membrane protein type by collocated hydrophobic amino acid pairs. J Comp Chem.

[B51] Liu H, Setiono R (1996). A probabilistic approach to feature selection – a filter solution. Proceedings of the 13th International Conference on Machine Learning.

[B52] Kohavi R, John GH (1997). Wrappers for feature subset selection. Artificial Intelligence.

[B53] John GH, Langley P (1995). Estimating Continuous Distributions in Bayesian Classifiers. Proceedings of the 11th Conference on Uncertainty in Artificial Intelligence.

[B54] Baldi P, Brunak S, Chauvin Y, Andersen CA, Nielsen H (2000). Assessing the accuracy of prediction algorithms for classification: an overview. Bioinformatics.

[B55] Rost B, Eyrich VA (2001). EVA: large-scale analysis of secondary structure prediction. Proteins.

[B56] Birzele F, Kramer S (2006). A new representation for protein secondary structure prediction based on frequent patterns. Bioinformatics.

[B57] Hutchinson EG, Thornton JM (1994). Revised set of potentials for beta-turn formation in proteins. Protein Sci.

